# ZBTB32 restrains antibody responses to murine cytomegalovirus infections, but not other repetitive challenges

**DOI:** 10.1038/s41598-019-51860-z

**Published:** 2019-10-24

**Authors:** Arijita Jash, You W. Zhou, Diana K. Gerardo, Tyler J. Ripperger, Bijal A. Parikh, Sytse Piersma, Deepa R. Jamwal, Pawel R. Kiela, Adrianus C. M. Boon, Wayne M. Yokoyama, Chyi S. Hsieh, Deepta Bhattacharya

**Affiliations:** 1 Department of Pathology and Immunology, Washington University School of Medicine, Saint Louis, Missouri 63110 United States of America; 2 Department of Medicine, Washington University School of Medicine, Saint Louis, Missouri 63110 United States of America; 3 Division of Rheumatology, Washington University School of Medicine, Saint Louis, Missouri 63110 United States of America; 4 Department of Immunobiology, University of Arizona College of Medicine, Tucson, AZ 85724 USA; 5 Department of Pediatrics, University of Arizona College of Medicine, Tucson, AZ 85724 USA; 6 Division of Infectious Diseases, Washington University School of Medicine, Saint Louis, Missouri 63110 United States of America; 7 Department of Molecular Microbiology, Washington University School of Medicine, Saint Louis, Missouri 63110 United States of America

**Keywords:** Immunological memory, Antimicrobial responses

## Abstract

ZBTB32 is a transcription factor that is highly expressed by a subset of memory B cells and restrains the magnitude and duration of recall responses against hapten-protein conjugates. To define physiological contexts in which ZBTB32 acts, we assessed responses by *Zbtb32*^−/−^ mice or bone marrow chimeras against a panel of chronic and acute challenges. Mixed bone marrow chimeras were established in which all B cells were derived from either *Zbtb32*^−/−^ mice or control littermates. Chronic infection of *Zbtb32*^−/−^ chimeras with murine cytomegalovirus led to nearly 20-fold higher antigen-specific IgG2b levels relative to controls by week 9 post-infection, despite similar viral loads. In contrast, IgA responses and specificities in the intestine, where memory B cells are repeatedly stimulated by commensal bacteria, were similar between *Zbtb32*^−/−^ mice and control littermates. Finally, an infection and heterologous booster vaccination model revealed no role for ZBTB32 in restraining primary or recall antibody responses against influenza viruses. Thus, ZBTB32 does not limit recall responses to a number of physiological acute challenges, but does restrict antibody levels during chronic viral infections that periodically engage memory B cells. This restriction might selectively prevent recall responses against chronic infections from progressively overwhelming other antibody specificities.

## Introduction

Immunological memory is a hallmark characteristic of the adaptive immune system. Upon clearance of infections or vaccines, a pool of antigen-specific lymphocytes remains, poised to rapidly respond to immunogen re-exposure. When these memory lymphocytes are subsequently engaged by their cognate immunogens, the resulting recall responses are typically of greater magnitude and rapidity than the antecedent primary reaction. A number of studies have identified gene expression differences between naïve and memory lymphocyte subsets^1–6^. However, the crucial genetic programs that functionally distinguish naïve and memory lymphocytes are not fully known, especially within the B cell lineage^7^.

The transcription factor ZBTB32 is highly expressed by a subset of mouse and human memory B cells, but not by naïve B cells^6,8–11^. We recently demonstrated that in the absence of ZBTB32, recall responses to T cell-dependent model antigens were much more rapid and durable than those mounted by control memory B cells^8^. In contrast, primary responses were unaffected by ZBTB32-deficiency against these hapten-protein conjugates. ZBTB32-deficient recall responses were characterized by the rapid production of antibody-secreting cells in the spleen, and then durable maintenance of plasma cells in the bone marrow^8^. ZBTB32 itself was not detectably expressed in these secondary plasma cells^8^. Instead, the direct effects of this transcription factor likely occurred during memory B cell activation, perhaps by limiting MHCII expression, antigen processing, and as a result, T cell help^8,12^. In the absence of ZBTB32, the resultant secondary plasma cells display enhanced transcriptional signatures of mitochondrial function^8^, which is critical for long-term plasma cell survival^13,14^.

Left unaddressed by our previous work was an etiological reason for having a specific negative regulator of memory B cell recall responses and the physiological consequences of lacking this factor. We first considered the possibility that ZBTB32 acts as a tumor suppressor of multiple myeloma, a plasma cell malignancy. Consistent with the possibility that ZBTB32 might play a role in malignancies, its expression distinguishes subtypes of diffuse large B cell lymphomas^15^. However, an examination of multiple myeloma sequencing studies and the Broad Institute Multiple Myeloma Genomics Portal (http://portals.broadinstitute.org/mmgp/home) revealed no evidence of recurrent *Zbtb32* mutations^16^. We next considered the possibility that inherited *Zbtb32* mutations may predispose towards autoimmunity. Again, a survey of genome-wide association study databases and primary literature revealed no connections between *Zbtb32* and autoimmune disorders^17–26^. Moreover, we observed no signs of spontaneous autoimmunity in *Zbtb32*^−/−^ animals. Finally, we reasoned that ZBTB32 might maintain the breadth of humoral immunity by preventing the unnecessary accumulation of secondary plasma cells. Most individuals bear 8–12 chronic viral infections that can become periodically reactivated under appropriate conditions^27^. This reactivation triggers memory recall responses, an inflation of oligoclonal specificities, and loss of lymphocyte diversity that becomes most pronounced with age^28^. Cytomegalovirus is perhaps the best studied pathogen in which each of these effects is observed, at least for T cells^29,30^. It is possible that ZBTB32 prevents or slows such effects for B cells. Reciprocally, if ZBTB32-deficiency prevents secondary plasma cells from dying, each recall response might leave behind an outsized footprint and competitively inhibit both pre-existing and subsequent plasma cells from accessing limited survival factors or ‘niches’^31^. We reasoned that chronic infections would be most likely to reveal such effects of ZBTB32 deficiency on memory B cell activation and plasma cell survival.

## Results

### ZBTB32 restrains MCMV-specific antibody responses after infection

ZBTB32 is highly expressed by isotype-switched memory B cells, both in mice and humans^6,8,9,11^. In mice, the highest levels of ZBTB32 are restricted to the CD80+ subset of memory B cells, which rapidly differentiate into plasma cells but lack the ability to initiate germinal center reactions^6^. Our studies using knockout mice demonstrated that ZBTB32 limits the rapidity and duration of memory B cell responses against hapten-protein conjugates^8^. Yet the physiological contexts in which this negative regulatory pathway is engaged remain unclear.

Murine cytomegalovirus (MCMV) infections trigger a slow but progressive inflation of antigen-specific IgG over time^32^. This inflation is not associated with increases in MCMV-specific IgM from naïve B cells, antibody avidity, or persistent germinal center reactions, implying the reactivation of isotype-switched memory B cells as MCMV intermittently exits latency^32^. As MCMV initiates a lifelong infection^33^, this process might continue for the lifespan of the host as the virus periodically becomes reactivated. We hypothesized that ZBTB32 limits the magnitude of this antibody inflation caused by MCMV infection.

**Figure 1:**
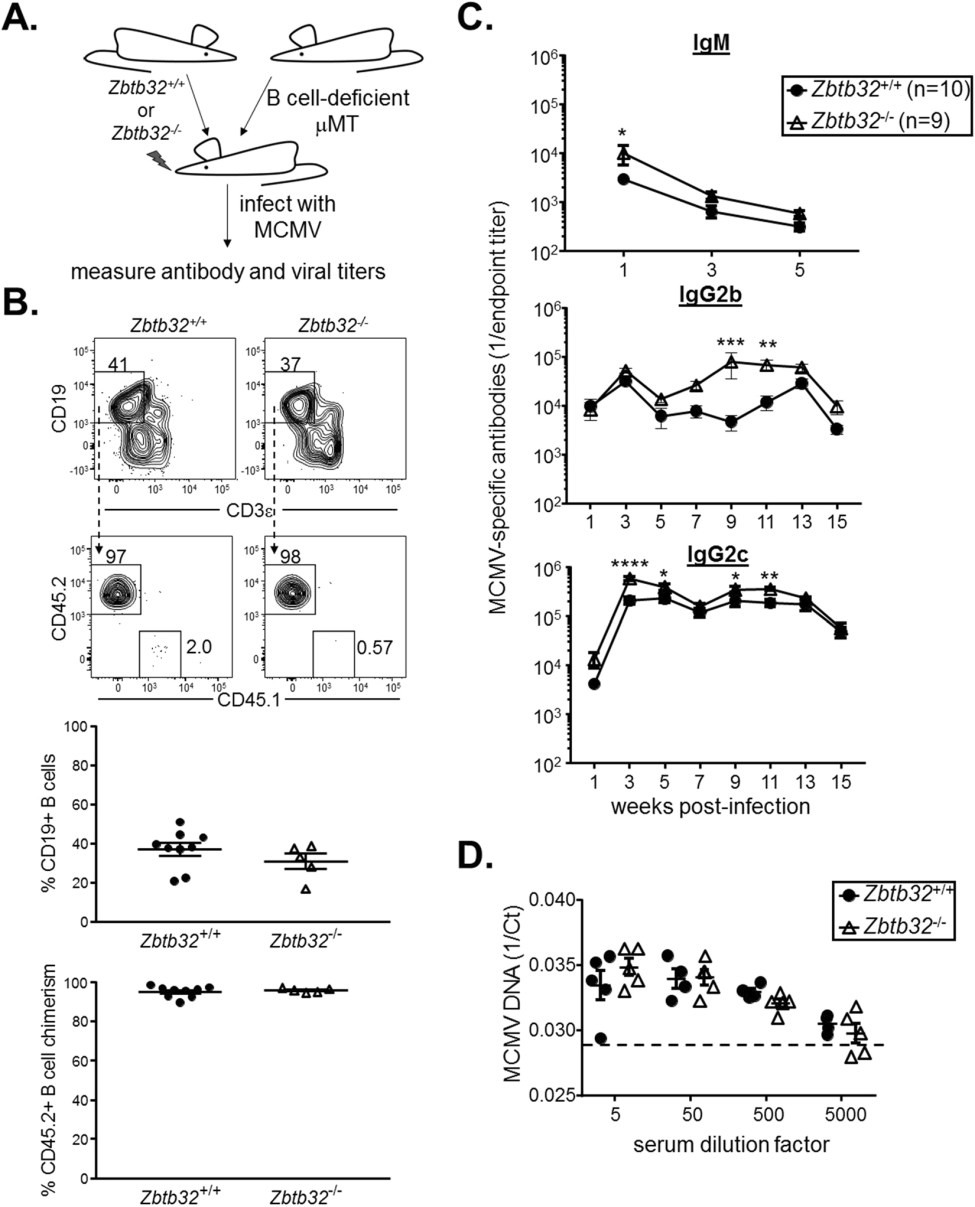
ZBTB32 restrains MCMV-specific antibody responses after infection. (**A**) Schematic of bone marrow chimeras and MCMV infections. Equal numbers of bone marrow cells from *Zbtb32*^−/−^ or *Zbtb32*^+/+^ donors and μMT donors were mixed and transplanted into irradiated wild-type IgH^a^ recipients. Chimeras were infected with MCMV-Smith and antigen-specific antibodies were followed over time. (**B**) Representative flow cytometry plots and quantification of peripheral blood B cell reconstitution in *Zbtb32*^+/+^ or *Zbtb32*^−/−^ bone marrow chimeras 8 weeks post-transplantaton. No significant differences were observed by students’ 2-tailed t-tests. (**C**) ELISA measurements of MCMV-specific IgM, IgG2b, and IgG2c over time. Mean values ± SEM are shown. *p < 0.05; **p < 0.005; ***p < 0.0005 by 2-way ANOVA and post-hoc Sidak’s multiple correction test. Data are cumulative of two independent experiments. (**D**) Serum MCMV viral titers as measured by quantitative RT-PCR. Threshold cycle (Ct) values were calculated at different serum dilutions at 1 week post-infection. Dashed line represents the value from uninfected naïve mice and the lower limit of detection. Mean values ± SEM are shown. No significant differences were observed by 2-way ANOVA and post-hoc Sidak’s multiple correction.

ZBTB32 has been shown to promote natural killer (NK) cell proliferation after MCMV infection^34^, and NK cells are essential for control of MCMV infection^35–37^, which would confound the study of ZBTB32 effects on B cells in genetically deficient animals. We therefore established mixed chimeras in which CD45.2+ bone marrow cells from *Zbtb32*^−/−^ or *Zbtb32*^+/+^ controls were mixed with equal numbers of B cell-deficient μMT bone marrow cells and transplanted into irradiated CD45.1+ recipients (Fig. 1A)^38^. After reconstitution, B cells were exclusively CD45.2+, and comparable levels of B cells were observed between *Zbtb32*^+/+^ and *Zbtb32*^−/−^ chimeras (Fig. 1B). This system ensures that while all B cells in the experimental group are derived from *Zbtb32*^−/−^ donors, half of all other hematopoietic and immune lineages, including NK cells, are derived from μMT mice and functionally normal within the same recipients (Fig. 1A).

Chimeras were challenged with 10^5^ PFU of MCMV-WT1, a subclone of the Smith strain of MCMV^39^, and antigen-specific antibody titers were measured at 1 week post-infection and every 2 weeks afterwards. IgM titers were slightly elevated in *Zbtb32*^−/−^ chimeras at week 1 (Fig. 1C, top panel). After this point, MCMV-specific IgM gradually declined irrespective of genotype and eventually fell below the limit of detection after week 5 (Fig. 1C, top panel). At weeks 1–3 post-infection, MCMV-specific IgG2b levels were similar in *Zbtb32*^−/−^ chimeras relative to *Zbtb32*^+/+^ controls (Fig. 1C, middle panel). Following a decline at week 5, presumably the point at which acute infection was resolved, MCMV-specific IgG2b levels rapidly rose in *Zbtb32*^−/−^ chimeras until week 9, reaching nearly 20-fold higher titers than in control chimeras (Fig. 1C, middle panel).

Between weeks 9 and 11 post-infection, MCMV-specific IgG2b levels plateaued in *Zbtb32*^−/−^ chimeras, whereas antigen-specific IgG2b rose in wild-type chimeras (Fig. 1C, middle panel). Yet despite this late increase, MCMV-specific antibodies from wild-type chimeras still remained below the levels observed in *Zbtb32*^−/−^ chimeras (Fig. 1C, middle panel). Similarly, antigen-specific IgG2c levels in *Zbtb32*^−/−^ chimeras rose to 3-fold higher levels than controls at week 3 post-infection, and then were maintained at modestly elevated levels through week 11 (Fig. 1C, bottom panel). After this point, antibody titers declined to the levels observed in wild-type chimeras (Fig. 1C, bottom panel). These data demonstrate that ZBTB32 restricts antibody responses to MCMV, particularly during the period after the initial acute response.

Although half of all non B-lineage cells in our chimeras were functionally wild-type, ZBTB32 has been implicated in both NK and T cell function^34,40–43^. Thus, we were concerned that MCMV viral levels might have differed between our control and experimental groups since half of all T and NK cells were ZBTB32-deficient. Elevated MCMV levels could in turn inflate antigen load and antibody responses. To test this possibility, we assessed MCMV levels through quantitative PCR. Serum levels of MCMV DNA were similar between ZBTB32-deficient and –sufficient chimeras at week 1 post-infection (Fig. 1D), arguing against differences in viral load. At later timepoints, MCMV was undetectable in the serum (not depicted), consistent with the establishment of latency. Coupled with our observation that initial IgG titers were similar between groups, our data demonstrate that ZBTB32-deficient chimeras show exaggerated MCMV-specific antibody levels despite similar viral loads as in controls.

### Initial activation stimulus, rather than isotype, dictates memory B cell dependence on ZBTB32

**Figure 2:**
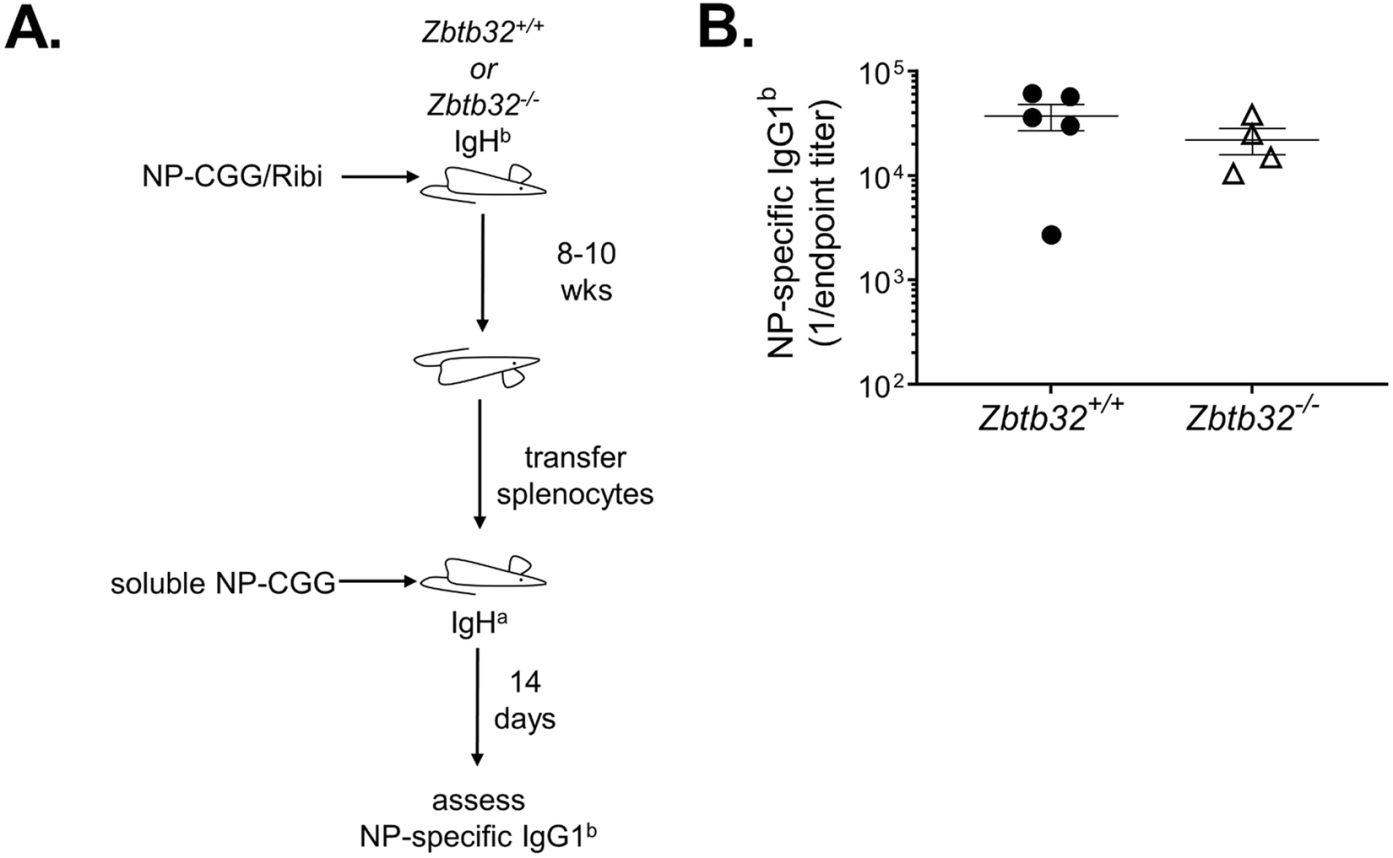
Ribi-adjuvanted immunization yields ZBTB32-independent recall responses. (**A**) Schematic of primary immunization, adoptive transfer, and secondary immunizations. (**B**) ELISA measurement of memory B cell responses. *Zbtb32*^+/+^ and *Zbtb32*^−/−^ mice were immunized Ribi-adjuvanted NP-CGG and 8–10 weeks later splenocytes were transferred to naïve *IgH*^*a*^ recipients. One day later, recipients were immunized intravenously with soluble NP-CGG. Donor IgG1^b^ NP-specific antibodies were quantified by ELISA. Each data point represents an individual mouse. Error bars depict geometric means ± 95% confidence interval. Differences were statistically insignificant as determined by Mann–Whitney test (p = 0.41).

In the absence of ZBTB32, IgG2b responses to MCMV were more dramatically affected than were IgM or IgG2c (Fig. 1C). There are several potential mechanisms for this observation. First, it is possible that only certain isotypes of memory B cells depend on ZBTB32 to restrain recall responses. Second, it may be that the initial innate activation stimuli dictate ZBTB32 dependence, irrespective of isotype. The latter mechanism would be similar to our findings on the related transcription factor, ZBTB20^44^. In these earlier studies, we determined that the adjuvant, rather than antibody isotype, determines the dependence of plasma cell survival on ZBTB20^44^. For ZBTB32, our previous work relied exclusively on alum-adjuvanted immunizations with NP-CGG^8^. We therefore immunized *Zbtb32*^+/+^ or *Zbtb32*^−/−^ mice with NP-CGG adjuvanted with monophosphoryl lipid A, a TLR4 ligand, and trehalose dicorynomycolate, a TLR2 ligand derived from Mycobacterium tuberculosis, in an oil-in-water emulsion. This formulation is similar to Ribi adjuvant, for which in contrast to alum, the bulk of long-term antibody production is TLR dependent^45^. At 8–10 weeks post-immunization, splenocytes were adoptively transferred into naïve IgH^a^ recipients (Fig. 2A). These mice were challenged with soluble NP-CGG to selectively recall donor memory B cells, and IgG1 responses were measured 2 weeks later. No differences in NP-specific IgG1^b^ were observed, irrespective of genotype (Fig. 2B). These data contrast with experiments when memory B cells were transferred after alum-adjuvanted immunization, where *Zbtb32*^−/−^ donor cells mounted a markedly higher and persistent IgG1 recall response than did controls beginning at day 3 post-immunization^8^. Thus, we conclude that the initial activation stimulus in the primary response that gives rise to memory B cells dictates ZBTB32-dependence, whereas the antibody isotype does not.

### ZBTB32-deficiency minimally impacts IgA responses to intestinal bacteria

**Figure 3:**
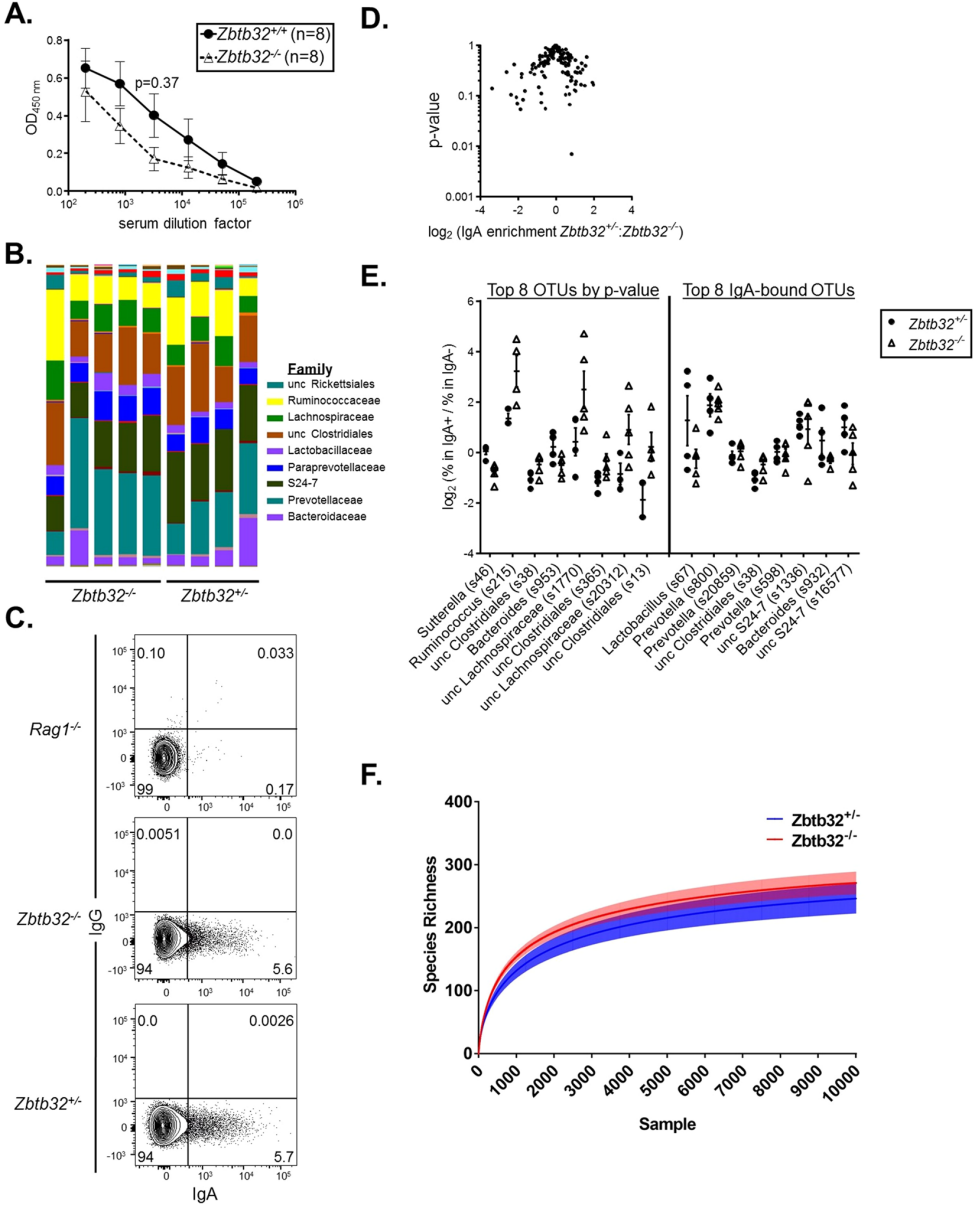
ZBTB32-deficiency minimally impacts IgA responses to intestinal bacteria. (**A**) Serum IgA levels in 8 week-old *Zbtb32*^−/−^ or *Zbtb32*^+/+^ mice, measured by ELISA. Mean values ± SEM are shown. No significant differences were observed by 2-way ANOVA followed by post-hoc Sidak’s multiple comparisons test. (**B**) Bacterial content in fecal pellets of *Zbtb32*^−/−^ and *Zbtb32*^+/−^ littermates as revealed by 16S rDNA sequencing. Data shown are family level taxa for individual mice. No statistically-significant differences between genotypes were observed at the family or OTU level by permutation ANOVA. (**C**) Representative flow cytometric plot of IgA-bound bacteria in fecal pellets. Bacteria were gated as DAPI+ and isotype control negative events and assessed for IgA and IgG staining. Data are representative of two independent experiments comparing littermates (*Zbtb32*^−/−^, n = 5; *Zbtb32*^+/−^, n = 4). (**D**) Volcano plot showing the IgA-enrichment (log2 (% of OTU in IgA+/IgA−) vs t-test p-value. As ratios are very susceptible to small denominators, data are calculated using a filtered dataset (139 OTUs present in ≥2 samples for each genotype at >0.1% frequency). In addition, IgA enrichment per individual was arbitrarily capped at log_2_(50 or 1/50) to limit effects of small denominators. No OTU comparison passes FDR < 0.25. (**E**) IgA enrichment values for top 8 OTUs based on greatest differences between genotypes by p-value, and for those with largest relative abundance. Taxonomic assignments at the genus level for each OTU are shown if available. IgA-enrichment is calculated as per (**D**). (**F**) Rarefaction plot shows the average species diversity and 95% confidence limits at different sampling intervals. Unc, unclassified at the taxa level presented, with the best higher level assignment noted.

ZBTB32 is even more highly expressed by memory B cells in the intestine than those in the spleen^10^. A substantial fraction of IgA+ memory B cells in the gut respond to intestinal bacteria repeatedly over the course of a lifetime^46–49^, generating both short- and long-lived plasma cells^50^. The persistence of IgA plasma cells of a given specificity is limited in part by competition with other more recently formed antibody-secreting cells directed against other antigens^51^. We thus reasoned that ZBTB32, by restricting the persistence of secondary plasma cells, might promote IgA diversity in the gut, and thereby promote microbial homeostasis. To begin to test this possibility, we first examined serum IgA levels, which can grossly reflect differences in microbial content^52^. Serum IgA levels trended slightly lower in *Zbtb32*^−/−^ animals relative to controls (Fig. 3A), but this did not reach statistical significance (2-way ANOVA, p = 0.37 at a serum dilution of 1:3200, powered to reveal 2.5-fold differences). To more precisely quantify bacterial composition, we performed 16S ribosomal RNA gene sequencing of fecal matter. Microbial content between *Zbtb32*^−/−^ and *Zbtb32*^+/−^ littermates was similar both at the operational taxonomic unit (OTU) (p = 0.211) and family level (p = 0.213) as determined by permutation-based ANOVA (Fig. 3B).

Having confirmed similar intestinal microbial content between *Zbtb32* genotypes, we next defined the specificities of IgA responses using Bacteria FACS. In this approach, IgA-bound bacteria from fecal matter are purified by fluorescence-activated cell sorting and subjected to 16S ribosomal RNA gene sequencing (Fig. 3C)^49,53,54^. The sequencing information allows for assignment of operational taxonomic units (OTUs) and an estimation of the diversity of bacteria recognized by IgA^49^. 16S sequencing of these IgA-bound bacteria revealed similar overall IgA specificities between *Zbtb32*^−/−^ and *Zbtb32*^+/−^ littermates (Fig. 3D), as assessed by the ratio of the OTU frequency in the IgA^+^ over the IgA^−^ sorted samples (2-way ANOVA, p = 0.26). There was one Sutterella OTU which was decreased in the knockout (t-test, p = 0.007), but was not significant after false discovery rate (FDR) correction, even using a filtered dataset of 139 OTUs (Fig. 3E, left panel). The top 8 IgA-bound OTUs in either genotype also did not show significant changes in the IgA-bound ratio (Fig. 3E, right panel). Finally, rarefaction plots, in which the number of different OTUs are plotted against the sequences per sample^55^, did not reveal statistically significant changes in the alpha diversity of IgA-bound OTUs between genotypes (Fig. 3F). While additional samples might provide more statistical power to demonstrate subtle differences in IgA binding to select OTUs, these data suggest that ZBTB32-deficiency does not markedly affect the specificity or production of IgA to gut bacteria.

**Figure 4:**
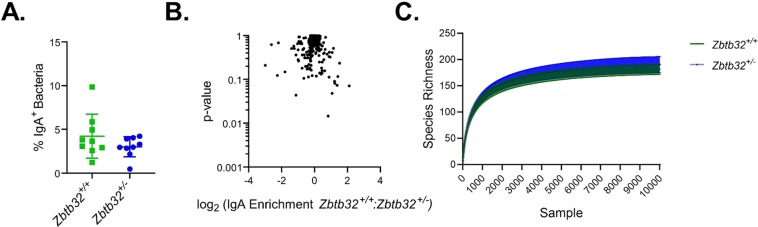
ZBTB32-haploinsufficiency minimally impacts IgA responses to intestinal bacteria. (**A**) Quantification of IgA-bound bacteria in fecal pellets. Bacteria were gated as DAPI+ and isotype control- events and assessed for IgA and IgG staining as in Fig. 3C. Lack of statistical significance was determined by students’ 2-tailed t-test. (**B**) Volcano plot showing IgA-enrichment (log2 (% of OTU in IgA+/IgA−) vs t-test p-value. No OTU comparison passed FDR < 0.25. (**C**) Rarefaction plot shows the average species diversity and 95% confidence limits at different sampling intervals.

Previous studies on NK cells demonstrated a significant phenotype in heterozygous *Zbtb32*^+/−^ mice relative to wild-type controls^34^. As our studies focused on comparisons between *Zbtb32*^−/−^ and *Zbtb32*^+/−^ mice, it is possible that we overlooked a dosage effect for ZBTB32. We therefore generated new crosses to compare bacterial IgA responses between *Zbtb32*^+/−^ and *Zbtb32*^+/+^ littermates. We observed no differences between *Zbtb32*^+/−^ and *Zbtb32*^+/+^ littermates in the frequency of IgA-bound bacteria (Fig. 4A). 16S sequencing of IgA-bound bacteria also revealed few statistically-significant differences in enriched OTUs between genotypes (Fig. 4B). Moreover, the overall diversity of IgA-bound OTUs was also similar between *Zbtb32*^+/−^ and *Zbtb32*^+/+^ littermates (Fig. 4C). We thus conclude that despite some differences in individual bacterial specificities, ZBTB32-deficiency does not limit overall IgA target diversity in the gut.

### ZBTB32 does not restrain recall responses to influenza vaccines

Primary responses to unadjuvanted influenza vaccines are often quite weak^56,57^. In contrast, memory B cells are capable of mounting antibody responses against both influenza infections and unadjuvanted vaccinations. For example, protective responses against the A/California/07/2009 H1N1 influenza strain were highly correlated with pre-existing cross-reactive memory B cells^58–61^. Upon heterologous challenges, these cross-reactive memory B cells might undergo further affinity maturation to focus the response on the new strain, both against the variable head region and conserved stalk region of influenza hemagglutinin (HA)^59^. The duration of influenza immunity after recall challenges can in some cases be very durable^62^, but is generally short-lived when unadjuvanted vaccines are used^63^.

**Figure 5:**
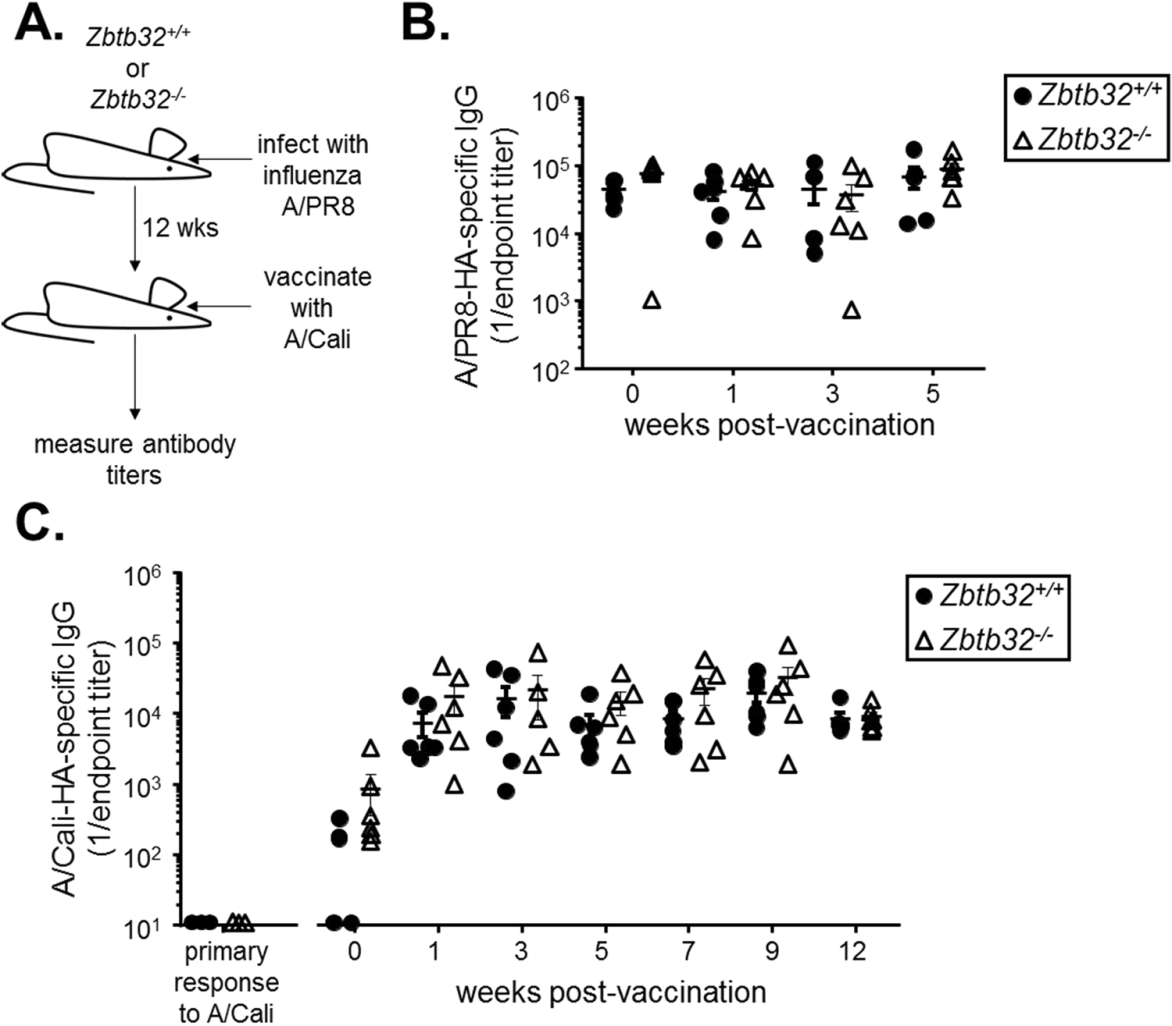
ZBTB32 does not restrain recall responses to influenza vaccines. (**A**) Schematic showing infection and heterologous vaccination schedule. (**B**) ELISA titers against recombinant hemagglutinin from A/PR8 at 12 weeks post-primary infection (0 weeks post-rechallenge), and after heterologous A/Cali vaccination. Mean endpoint titers ± SEM are shown. No statistically significant differences were observed by Mann-Whitney test, or by 2-way ANOVA and post-hoc Sidak’s multiple comparison’s test. (**C**) ELISA titers against recombinant hemagglutinin from A/California after vaccination of naïve mice (left panel) or A/PR8-immune mice (right panel). Mean endpoint titers ± SEM are shown. No statistically significant differences were observed by Mann-Whitney test, or by 2-way ANOVA and post-hoc Sidak’s multiple comparison’s test.

To determine if ZBTB32-deficiency extends the duration of antibody responses against influenza, we adopted an infection and heterologous vaccination model (Fig. 5A). *Zbtb32*^−/−^ or control littermates were first infected with the influenza H1N1 strain A/Puerto Rico/8/1934 (A/PR8). Antibody responses were similar against recombinant A/PR8 hemagglutinin (HA) protein between *Zbtb32*^+/+^ and *Zbtb32*^−/−^ littermates through 12 weeks post-infection (Fig. 5B). At this point, mice were re-challenged with H1N1 A/California/7/2009 (A/Cali) monovalent vaccine (Fig. 5A). Naïve mice that had not first been infected with A/PR8 mounted no detectable antibody responses to HA from A/Cali (Fig. 5C, left column), confirming that primary responses to this unadjuvanted vaccine are weak. Moreover, A/PR8-immune mice showed minimal serum antibody reactivity against A/Cali HA prior to vaccination (Fig. 5C, week 0). Vaccination with A/Cali did not enhance antibody responses to A/PR8 HA (Fig. 5B, weeks 1–5). However A/PR8-immune mice mounted a robust recall response against A/Cali HA (Fig. 5C), presumably because of the presence of cross-reactive memory B cells. These data are consistent with our previous findings that long-lived plasma cells, which durably maintain antibodies in the serum, possess specificities restricted to the original strain, whereas memory B cells are considerably more diverse^64^. In this system, secondary responses to A/Cali were exceptionally durable even in wild type mice, with no measurable decline in antibody titers between 1–12 weeks post-vaccination (Fig. 5C). Recall responses by ZBTB32-deficient mice were similar to controls throughout the course of the experiment (Fig. 5C). Thus, unlike the transient responses elicited by hapten-protein conjugate-based booster immunizations^8^, antigen-specific antibody titers are already maintained durably after an acute influenza vaccination and ZBTB32 exerts no additional influence in restricting responses.

## Discussion

The adaptive immune system is exposed to nearly constant stimulation, both from new antigens as well as immunogens that have been seen previously. Memory lymphocytes are responsible for responding to previously encountered antigens, and generally mount more rapid and robust responses than their naïve precursors. In previous work using model antigens, we demonstrated that the expression of ZBTB32 is utilized by memory B cells to attenuate recall responses^8^. On one hand, the existence of such a negative regulator seems peculiar. Durable and robust recall responses seem to be traits that should be evolutionarily favored. On the other hand, there are presumably only a finite number of plasma cells that can be maintained^31^. Thus, mechanisms may exist to limit the numbers of long-lived plasma cells to only what is necessary for protection^65^. Indeed, we previously demonstrated that only 1 μl of passively transferred immune serum was sufficient to protect mice from an otherwise lethal dose of West Nile virus^64^. This corresponds to antibodies from only 3 antigen-specific long-lived plasma cells. Thus, keeping a relatively small number of antigen-specific plasma cells would maximize the potential breadth of antibodies while simultaneously maintaining protective immunity.

The contexts in which memory B cells would need to engage such a negative regulatory pathway, however, are unclear. Influenza and malaria infections can occur repeatedly^62,66^, which in turn could reduce the overall diversity of plasma cells if factors such as ZBTB32 were not in place. Yet the frequency of these types of infections, even in endemic areas, are unlikely to exceed one per year^66^. Thus, it seems unlikely that recall responses to these acute infections would dramatically alter the content of pre-existing plasma cells, even in the absence of ZBTB32. Indeed, based on our previous work using high doses of hapten-based antigens^8^, we estimate that only 1000–2000 long-lived plasma cells were generated in excess and maintained following recall responses by ZBTB32-deficient mice. Thus, repeated and distinct acute challenges seem unlikely to necessitate negative regulatory mechanisms such as ZBTB32 expression. Indeed, we observed no role for ZBTB32 in restricting antibody responses in mouse models of influenza infection and vaccination, nor in responses to intestinal bacteria. The mechanistic basis for the differences in responses to haptens, influenza viruses, and intestinal bacteria is not clear, but may involve the innate signals and inflammatory environment that imprint upon the memory B cell population^67^. In support of this concept, memory B cells of different antibody isotypes use distinct transcription factors to form and persist^68^. Immunoglobulin isotype-switching is linked to the particular cytokines induced by immunization- or infection-specific innate signals^69^. Such differences in innate signals during memory B cell ontogeny could in turn influence the persistence of secondary plasma cells following recall responses. Indeed, our previous work on a related BTB-POZ transcription factor, ZBTB20, revealed an adjuvant-specific requirement in mediating durable antibody responses^44^. Nonetheless, both for ZBTB20 and ZBTB32, our findings argue against a direct role for the antibody isotype per se. Rather, we propose that the initial activation stimuli and resultant Tfh cells determine the dependence on ZBTB20 and ZBTB32 for controlling antibody responses.

Responses to chronic infections may require distinct negative regulatory mechanisms to maintain homeostasis. CMV, in particular, has a dramatic effect on the mammalian immune system, triggering large oligoclonal expansions of memory lymphocytes with age^29,30^. There is no clear consensus on how these expansions impact responses to other pathogens^28^. Yet it seems likely that negative regulatory pathways would be important from keeping recall responses to chronic infections from completely overtaking both the naïve repertoire and pre-existing immunity. Mitigating these B cell recall responses would also be important given that antibodies are not especially protective against chronic infections such as CMV or norovirus^70,71^. Here, we found that ZBTB32 indeed restrains antibody responses against MCMV. This effect was most pronounced after the acute infection phase, and was more modest at the initial and late stages of infection. Though the end effect of ZBTB32-deficiency may not be large for any single infection, this pathway may be particularly important given that most humans are chronically infected by 8–12 different viruses^27^. Mice exposed to more physiological insults than those in specific pathogen-free environments are better mimics of the human experience^72^, and may further reveal the importance of ZBTB32 in limiting recall responses.

## Materials and Methods

### Ethics statement

All procedures in this study were specifically approved and carried out in accordance with the guidelines set forth by the Institutional Animal Care and Use Committee at Washington University (approval numbers 20160259 and 20160002) and the University of Arizona (approval number 17–266). Euthanasia was performed by administering carbon dioxide at 1.5 L/minute into a 7 L chamber until 1 minute after respiration ceased. After this point, cervical dislocation was performed to ensure death.

### Bone marrow chimeras and MCMV infection

Bone marrow cells harvested from *Zbtb32*^+/+^ and *Zbtb32*^−/−^ mice between 8–10 weeks of age were mixed with that from μMT mice at a 1:1 ratio and injected intravenously into 800-cGy-irradiated CD45.1 recipients. After 8 weeks, peripheral blood was sampled from the tail vein to confirm reconstitution. The recipients were then infected intraperitoneally (i.p) with 1 × 10^5^ PFU of MCMV-WT1, a subclone of the Smith strain of MCMV^39^, amplified in salivary glands, and mice were bled at the indicated time points to assess MCMV-specific IgM, IgG2c and IgG2b in the serum. ELISA plates were coated overnight at 4 °C with 1 × 10^3^ PFU/ml of tissue-culture propagated, plaque-purified wild type MCMV in bicarbonate coating buffer (0.1 M sodium bicarbonate and 0.02% sodium azide at pH 9.6). Plates were washed with wash buffer (PBS containing 0.05% Tween 20) and after blocking 1 hr with PBS supplemented with 2% BSA and 0.05% Tween 20 at 37 °C, serially diluted serum samples were added and incubated for 1 h at room temperature. Technical duplicates were performed for every serum sample. Plates were washed with PBS with 0.05% Tween 20 and incubated with 1 µg/ml biotinylated antiIgM, anti-IgG2c or anti-IgG2b for 1 hr followed by streptavidin conjugated horseradish peroxidase (HRP) for 45 min. Peroxidase activity was detected by tetramethylbenzidine (Dako) substrate and the reaction was quenched with 2 N H_2_SO_4_ and optical densities were quantified at 450 nm. The endpoint titer of each sample was determined using Prism software (GraphPad Software) from a one phase exponential decay curve defined as the dilution that generates an OD_450_ value of the background plus 3 SD.

### Immunization and adoptive transfer for recall responses

*Zbtb32*^+/+^ and *Zbtb32*^−/−^ mice 8–10 weeks of age were immunized intraperitoneally (i.p.) with a single dose of 100 μg NP-CGG (hapten protein ratio: 15–22; Biosearch Technologies) adjuvanted with 100 μl Sigma Adjuvant System (Ribi). Spleens were harvested 8–10 weeks post immunization and single cell suspensions of splenocytes were subjected to gradient centrifugation using Histopaque 119 (Sigma-Aldrich) for 10 min at 2000 × g to remove non-cellular debris. Interface cells were then collected and red blood cells were lysed by resuspending in buffer containing 0.15 M NH_4_Cl, 10 mM KHCO_3_, 0.1 mM EDTA, pH 7.2. Cells were washed twice with PBS and 10% of the cells were retained for flow cytometric analysis. The remaining splenocytes were adoptively transferred into non-irradiated B6.Cg*Igh*^*a*^*Thy1*^*a*^*Gpi1*^*a*^ (*IgH*^*a*^) recipient mice (Jackson Labs). A recall response was then elicited in recipient mice 24 hours later by intravenous administration of 50 μg of soluble unadjuvanted NP-CGG.

### Serological analysis for recall responses

ELISA plates were coated overnight at 4 °C with 5 µg/ml of NP_16_bovine serum albumin (BSA) in bicarbonate coating buffer (0.1 M sodium bicarbonate and 0.02% sodium azide at pH 9.6). Plates were washed with wash buffer (PBS containing 0.05% Tween 20) and after blocking 1 hr with blocking buffer (PBS supplemented with 2% BSA and 0.05% Tween 20) at 37 °C, serially diluted serum samples were added and incubated for 1 h at room temperature. Technical duplicates were performed for every serum sample. Plates were washed with PBS with 0.05% Tween 20 and incubated with 1 µg/ml biotinylated antiIgG1^b^ (B682, BD Biosciences) for 1 hr followed by streptavidin conjugated horseradish peroxidase for 45 min. Peroxidase activity was detected by tetramethylbenzidine substrate (Dako) and the reaction was quenched with 2 N H_2_SO_4_ and optical densities were quantified at 450 nm. The endpoint titer of each sample was determined using Prism software (GraphPad Software) from a one phase exponential decay curve defined as the dilution that generates an OD_450_ value of the background plus 3 standard deviations.

### MCMV quantification by qPCR

SYBR green-based real-time qPCR was performed to measure viral load in the serum. Briefly 10ul of serum was diluted in PBS to a final volume of 50 µl and heated at 95 C for 3 min and cooled rapidly on ice for 3 to 5 mins. 2 ul of serum was serially diluted and used to perform qRT-PCR using primers MCMV-IE1 Forward: 5′-AGCCACCAACATTGACCACGCAC-3′ and MCMV-IE1 Reverse: 5′-GCCCCAACCAGGACACACAACTC-3′^73^.

### Influenza infection, vaccination, and assessment of antibody titers

*Zbtb32*^+/+^ and *Zbtb32*^−/−^ mice between 8–10 weeks of age were infected intranasally with 0.01 × LD_50_ A/Puerto Rico/8/1934 virus and were re-challenged with 50 μl H1N1/A/California/7/2009 subunit vaccine (Novartis) by intramuscular injection 12 weeks post-infection. Coding sequences for HA from A/PR8 and A/California/7/2009 were cloned into pEF1α myc-His B (Invitrogen) and engineered to contain Y98F mutations as previously described^74^. HEK293T cells were transfected with these constructs in 10 cm^2^ dishes using FuGene HD (Promega) and supernatants collected every 24 hours from days 2–6. Recombinant HA was purified over Ni-NTA columns (GE Health Sciences) according to manufacturer’s instructions. ELISA plates were coated overnight at 4 °C with 50 µg/ml of purified PR8-HA or Cal-HA protein in bicarbonate coating buffer (0.1 M sodium bicarbonate and 0.02% sodium azide at pH 9.6). ELISAs were performed as above, except with 1 µg/ml of horse radish peroxidase (HRP) conjugated anti-IgG was used to detect HA-specific antibodies irrespective of IgG isotype.

### Bacteria FACS

Stool samples were collected from 8 week old *Zbtb32*^−/−^ and *Zbtb32*^+/−^ control littermates. Samples were resuspended in sterile PBS by vortexing and treated with N-Acetyl-L-cysteine (Sigma) to disrupt the mucus network. Homogenized samples were passed through a 70 μm nylon filter and stained with DAPI (Sigma), anti-mouse IgA DyLight 650 (Abcam ab97014), anti-mouse IgG phycoerythrin (Jackson ImmunoResearch 115-115-164), and Goat IgG FITC Isotype control (Abcam ab37374). Samples were sorted on a BD FACS Aria IIu for IgA bound bacteria.

16S rDNA was amplified using the standard protocol from Caporaso *et al*.^75^. High throughput sequencing was performed on the Illumina Miseq platform. Assignment of bacterial OTUs from 16S sequences was accomplished using UParse OTU clustering algorithm and taxonomy assigned via QIIME using the uclust method with greenegenes database 13.8^76,77^. Further analysis of 16S sequencing was done on R statistical computing platform with the use of the Vegan package (https://cran.r-project.org/web/packages/vegan/vegan.pdf).

## Data Availability

The datasets generated during and/or analyzed during the current study are available from the corresponding author on reasonable request.
